# *Arabidopsis thaliana* nucleosidase mutants provide new insights into nucleoside degradation

**DOI:** 10.1111/j.1469-8137.2011.03711.x

**Published:** 2011-07

**Authors:** Heike Riegler, Claudia Geserick, Rita Zrenner

**Affiliations:** 1 Max-Planck-Institute of Molecular Plant Physiology14467 Potsdam, Germany; 2 Leibniz-Institute of Vegetable and Ornamental Crops14979 Grossbeeren, Germany

**Keywords:** *Arabidopsis thaliana* mutant, nucleoside degradation, nucleotide metabolism, uridine nucleosidase, xanthosine nucleosidase

## Abstract

- A central step in nucleoside and nucleobase salvage pathways is the hydrolysis of nucleosides to their respective nucleobases. In plants this is solely accomplished by nucleosidases (EC 3.2.2.x).
- To elucidate the importance of nucleosidases for nucleoside degradation, general metabolism, and plant growth, thorough phenotypic and biochemical analyses were performed using *Arabidopsis thaliana* T-DNA insertion mutants lacking expression of the previously identified genes annotated as uridine ribohydrolases (*URH1* and *URH2*).
- Comprehensive functional analyses of single and double mutants demonstrated that both isoforms are unimportant for seedling establishment and plant growth, while one participates in uridine degradation. Rather unexpectedly, nucleoside and nucleotide profiling and nucleosidase activity screening of soluble crude extracts revealed a deficiency of xanthosine and inosine hydrolysis in the single mutants, with substantial accumulation of xanthosine in one of them. Mixing of the two mutant extracts, and by *in vitro* activity reconstitution using a mixture of recombinant URH1 and URH2 proteins, both restored activity, thus providing biochemical evidence that at least these two isoforms are needed for inosine and xanthosine hydrolysis.
- This mutant study demonstrates the utility of *in vivo* systems for the examination of metabolic activities, with the discovery of the new substrate xanthosine and elucidation of a mechanism for expanding the nucleosidase substrate spectrum.

## Introduction

Nucleotides and nucleic acids were among the first spontaneously synthesized biomolecules (Powner *et al.*, 2009), and they play an important role in evolution, information preserving and catalytic activity (Joyce, 2002). While nucleotides are important intermediates in nucleic acid synthesis and energy metabolism, nucleotide sugars are necessary for storage compound biosynthesis and for the synthesis of structure-building compounds such as cellulose. In addition, purine nucleotides are involved in signal transduction and are components of cofactors participating in enzymatic reactions (e.g. NAD, FAD and CoA).

As an evolutionarily ancient and indispensable complex of metabolic pathways, the metabolism of both classes of nucleotides in plants is comparable to that in other organisms (Moffatt & Ashihara, 2002; Zrenner *et al.*, 2006). It starts with the formation of central metabolites, while all purines are derived from IMP (van der Graaff *et al.*, 2004) and all pyrimidines from UMP (Giermann *et al.*, 2002; Schröder *et al.*, 2005). The whole range of nucleotides is then generated by amination, desamination and oxidation of the heterocyclic ring system.

There are also central pathways for degradation, with all pyrimidines being degraded via the nucleoside uridine, and all purines via the nucleobase xanthine. Thus, xanthine dehydrogenase is the bottleneck in purine degradation (Hesberg *et al.*, 2004). Reducing the capacity of xanthine dehydrogenase causes severe phenotypes such as growth retardation, early senescence and infertility (Nakagawa *et al.*, 2007). By contrast, functional analysis of uracil catabolism has revealed that the role of pyrimidine degradation in growth and development in *Arabidopsis thaliana* is of only minor importance (Zrenner *et al.*, 2009). Mutants of pyrimidine degradation unable to catabolize [2-^14^C]-uracil did not show any difference from wild type when grown under standard growth conditions.

Compared with *de novo* synthesis, the salvage pathways provide an energy-saving recycling mechanism, where nucleosides or nucleobases are converted into the respective nucleotides. Nucleoside kinase activity and nucleobase phosphoribosyltransferase activity have been demonstrated for a variety of plants (Ashihara *et al.*, 2000; Katahira & Ashihara, 2002; Loukanina *et al.*, 2008). Mutants deficient in adenine phosphoribosyltransferase are male sterile (Gaillard *et al.*, 1998), while *A. thaliana* mutants affected in different uracil phosphoribosyltransferase genes display phenotypes of varying severity (Islam *et al.*, 2007; Mainguet *et al.*, 2009). In addition, increased availability of the co-substrate phosphoribosylpyrophosphate in the cytosol leads to enhanced uracil salvage and higher biomass accumulation in plants (Koslowsky *et al.*, 2008), thus demonstrating the importance of salvage reactions. To date, no mutant plants with altered uridine kinase activity have been reported, but mutants lacking adenosine kinase show developmental abnormalities and reduced transmethylation (Moffatt *et al.*, 2002).

While in animals interconversion of nucleosides and nucleobases is catalyzed by phosphorylases (EC 2.4.2.x), in plants only nucleosidase activity (EC 3.2.2.x) has been demonstrated (Katahira & Ashihara, 2002, 2006). Therefore, plants can be used as a superior research tool for functional analyses of nucleosidase activities. Recently, the importance of nucleoside hydrolysis has been rediscovered (Riewe *et al.*, 2008), and an *A. thaliana* enzyme catalyzing uridine hydrolysis was heterologously expressed by Jung *et al.* (2009). It carries the N-terminal finger print motif and additional sequence characteristics common to all nucleoside hydrolases (Versees & Steyaert, 2003). Transformants with altered expression of this nucleoside hydrolase indicate that this protein plays a crucial role in uridine degradation. Therefore, knock-out mutants with a complete loss of this protein may provide important insights into the salvage of pyrimidines. Other nucleoside-degrading enzymes have been purified and characterized from various plant species (Campos *et al.*, 2005; Szuwart *et al.*, 2006), but their genetic background is still unknown.

This work focuses on the investigation of mutant plants as a tool for functional analyses of nucleosidase activities to elucidate their importance for nucleoside degradation, general metabolism and plant growth. A thorough *in planta* biochemical investigation demonstrates the utility of this *in vivo* system for the study of metabolic activities, with the discovery of the new substrate xanthosine and elucidation of a mechanism for expanding the substrate spectrum.

## Materials and Methods

### Plant material and growth conditions

*Arabidopsis thaliana* L. Heynh Columbia-0 (Col), SALK_083120 (*URH1*) and SALK_128723 (*URH2*) were obtained from the Nottingham Arabidopsis Stock Centre (University of Nottingham, Loughborough, UK). Seeds were surface-sterilized and aseptically grown on half-strength MS medium including vitamins (Murashige & Skoog, 1962), 0.5% sucrose and 0.7% agar. Seeds were imbibed at 4°C in darkness (48 h) and grown in a 12-h photoperiod (photon flux density 100 μmol m^−2^ s^−1^; 21°C light; 18°C dark). After 3 wk, seedlings were transferred to soil and grown under a 8-h photoperiod (photon flux density 150 μmol m^−2^ s^−1^; 20°C light; 16°C dark).

### Determination of nucleoside hydrolase activity

Frozen plant material was powdered using a ball mill (Retsch, Haan, Germany). Then 100 mg of frozen material was mixed with 200 μl of cold extraction buffer (50 mM Hepes-NaOH (pH 7.6), 2 mM EDTA and 2 mM DTT) and kept at 4°C. The homogenate was centrifuged (11 000 ***g*** for 5 min) and the supernatant was desalted on spin columns using Sephadex G-25 fine (Amersham Pharmacia Biotech, Nümbrecht, Germany) with a 3-ml bed size equilibrated with 50 mM NaH_2_PO_4_/Na_2_HPO_4_ (pH 7.5). Protein content was determined using the Bio-Rad Protein Assay (Bio-Rad, Munich, Germany) and was 0.9 to 1.8 mg of soluble protein per g fresh weight for roots, and 3.5 to 5 mg of soluble protein per g fresh weight for leaves. Four time-points in the linear range of product formation (0, 5, 10 and 20 min) were used for each single activity determination, as follows: 35 μl of supernatant (10–100 μg of soluble protein) was added to 35 μl of reaction buffer (50 mM NaH_2_PO_4_/Na_2_HPO_4_ (pH 7.5) and 10–5000 μM nucleoside) and incubated at 30°C. Reactions were stopped by adding 20 μl of 16% trichloracetic acid. After neutralization with 3 μl of 10 M KOH and removal of the precipitate by centrifugation, the produced nucleobases were quantified by ion-pair reversed-phase HPLC (Zrenner *et al.*, 2009). For quantification of the residual activity, the pellet of the centrifuged homogenate was mixed with the initial amount of 50 mM NaH_2_PO_4_/Na_2_HPO_4_ (pH 7.5) and activity was determined as described above, while a shaker was used during the incubation procedure.

### Transient expression of URH-GFP fusion proteins

Entire *URH1* and *URH2* open reading frames were amplified from first-strand cDNA by PCR with Pfu-polymerase (MBI Fermentas, St. Leon-Rot, Germany) using oligonucleotides URH1_F 5′-CACCATGGATTGTGGTATGGAGAATTG, URH1_R 5′-TGGCTTCATCAGCTTTGCTTT, URH2_F 5′-CACCATGGCGATAGGAGACCGC, and URH2_R 5′-AGACTCCATAAGCCTATCCATTATGA. PCR products were inserted into the entry vector pENTR./SD/D-TOPO® (Invitrogen). Positive clones were either recombined into pET-DEST42 (Invitrogen) for C-terminal His-fusion and recombinant protein expression, or recombined into the pK7FWG2 plant transformation vector for C-terminal GFP fusion (Karimi *et al.*, 2002) and transferred into *A. thaliana* protoplasts (Yoo *et al.*, 2007). Protoplasts were analyzed using the laser scanning microscope Leica DM IRB at 488 nm (cytosolic control, beta-lactamase in pK7FWG2; pUC vector containing 35S promoter-driven expression of the first 80 amino acids of dihydroorotate dehydrogenase fused to DsRed (Giermann *et al.*, 2002) was used as mitochondrial control.

### Recombinant protein expression, purification and activity determination

Over-expression was achieved using single colonies of freshly transformed *Escherichia coli* strain BL21(DE3). To reduce the formation of inclusion bodies, 150 ml of uninduced overnight culture was diluted with 350 ml of fresh LB and cultivated at room temperature. When bacteria optical density *OD*_600_ reached 1.2, protein expression was induced with 0.5 mM Isopropyl ß-D-1-thiogalactopyranoside (IPTG) for a maximum of 3 h at room temperature. Recombinant proteins were purified using standard procedures with ice-cold lysis buffer containing 1 mM Phenylmethanesulfonylfluoride (PMSF) and Ni-nitrilotriacetic acid agarose (Ni-NTA) agarose under nondenaturing conditions (QIAexpressionist; Qiagen, Hilden, Germany). To obtain better purification results, 50 mM imidazol was included in the washing solution and recombinant proteins of the second eluate containing 300 mM imidazol were used in activity testing. While URH1 could be easily isolated with this procedure from soluble bacterial extracts, some of the URH2 proteins were always found in the insoluble residue after induction and extraction. However, the results obtained for URH2 using this procedure were better than those obtained using a variety of other protocols. Activity measurements were performed using desalted eluate fractions as described above with the respective nucleoside at 200 μM. For the URH1 + URH2 mixture, equal amounts of purified and desalted eluate fractions representing equimolar amounts of URH1 and URH2 were mixed.

### Isolation of mutants

Plants were obtained from the Salk collection (Alonso *et al.*, 2003). Screening and selection within mutant populations were carried out following the Signal Salk instructions (http://signal.salk.edu). Genomic DNA was isolated by a standard procedure using phenol-chloroform extraction from third-generation plants. PCR genotyping was performed using T-DNA LB-specific primers and gene-specific primer pairs of oligos urh1 left, 5′-TGCTCGCAAATGAACTATCG; urh1 right, 5′-ACAGACCCAGGAATTGGTGA; urh2 left, 5′-ACCAAAACTTCCCTCCACCT; urh2 right, 5′-GCTAGTTTGTCCTTGTCATCAG; and SALK LBb, 5′-GCGTGGACCGCTTGCTGCAACT. Homozygous mutants were isolated from selfed populations of the respective mutant. The sites of T-DNA insertions were verified by sequencing of border regions. Gene knock-out was confirmed by RT-PCR. Total RNA was isolated using NucleoSpin Plant II (Macherey-Nagel, Düren, Germany) and single-strand cDNA was synthesized using SuperScript III RNaseH^−^ reverse transcriptase (Invitrogen GmbH, Karlsruhe, Germany). Quantitative real-time PCR was performed for *URH1* using SYBR® Green and StepOnePlus System (Applied Biosystems, Darmstadt, Germany) and a temperature profile of 95°C for 10 min followed by 40 cycles of 95°C for 15 s and 60°C for 60 s. cDNA for *urh2* mutants was used as a PCR template with a temperature profile of 95°C for 5 min followed by 35 cycles of 95°C for 30 s, 58°C for 30 s and 74°C for 1 min.

### Metabolite analyses

Trichloroacetic acid/ether extracts from frozen powdered material were made and nucleotides and nucleotide sugars were measured by anion-exchange HPLC (Schröder *et al.*, 2005). Nucleosides and nucleobases were analyzed by ion-pair reversed-phase HPLC (Zrenner *et al.*, 2009). Unpaired two-tailed *t-*tests were used to compare pooled data from different types of material.

### [^14^C]-nucleoside metabolism

All tracer experiments were performed aseptically with whole plants. Seeds were surface-sterilized, imbibed for 2 d at 4°C and transferred to solid low-nitrogen medium (0.6 mM KH_2_PO_4_/K_2_HPO_4_ (pH 5.8), 0.75 mM MgSO_4_, 1.5 mM CaCl_2_, 9.8 mM KCl, 0.1 mM KNO_3_, 0.1 mM NH_4_NO_3_, micronutrients and vitamins according to Murashige & Skoog (1962) and 0.7% agar), at one seed per 12-ml Petri dish. Plants were grown for 6 wk under a 12-h photoperiod. For feeding, 500 μl (0.5 μCi) of ^14^C-precursor (0.2 μCi μmol^−1^ [2-^14^C]-uridine, 0.5 μCi μmol^−1^ [8-^14^C]-inosine and 0.2 μCi μmol^−1^ [8-^14^C]-xanthosine) was spread on the agar surface and each open plate was placed in a gastight box (Phytatray; Sigma-Aldrich, Munich, Germany) together with a 3MM Whatman filter paper soaked with 700 μl of 4M KOH. The incubation proceeded in the dark with one exchange of the filter after 24 h. After 48 h, plants were washed three times with water to remove possible surface contamination. After fresh weight determination, tissue was frozen in liquid nitrogen and powdered using a ball mill. Radiolabeled metabolites were extracted from 250 to 580 mg of material by homogenization in 500 μl of cold 10% perchloric acid containing 10 mM EGTA for 20 min on ice. After centrifugation at 11 000 ***g*** and 4°C for 5 min, the supernatant was removed and kept. The pellet was washed twice with 150 μl of 10% perchloric acid, and then all three supernatants were combined and neutralized using 5 M KOH containing 1 M triethanolamine. This supernatant fraction (supernatant1) contained unmetabolized nucleosides, catabolic pathway intermediates, nucleotides and nucleotide sugars, whereas the remaining pellet contained the insoluble DNA and RNA together with cell wall material and proteins. In certain cases, this pellet fraction was incubated for 20 h with 500 μl of 0.3 M KOH at 37°C with shaking based on a protocol of Ashihara (1977). After centrifugation at 11 000 ***g*** for 5 min, the supernatant was removed and kept. The pellet was washed with 100 μl of 0.3 M KOH, and both KOH supernatants were combined and neutralized (supernatant2). This supernatant2 fraction contained label that had been incorporated in the RNA. Radioactivity from the filter was eluted for 48 h in 7 ml of water. Radioactivity was determined using an LS6500 Multi-Purpose Scintillation Counter and Ready Safe scintillation cocktail (Beckman-Coulter, Krefeld, Germany).

## Results and Discussion

### Nucleoside metabolism in *Arabidopsis thaliana*

**Fig. 1:**
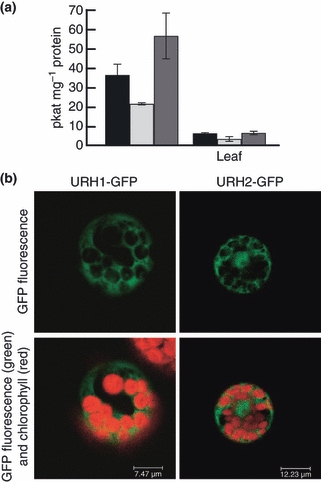
Nucleoside hydrolase activities and localization of nucleoside hydrolase (URH)-GFP fusion proteins. (a) Total activity of nucleoside to nucleobase conversion in soluble fractions of extracts from 10-wk-old *Arabidopsis thaliana* plants. Uridine hydrolase, black bars; inosine hydrolase, pale gray bars; xanthosine hydrolase, dark gray bars. Data are expressed in pkat per mg total soluble protein and show mean ± SD (*n* = 3). Substrate concentrations correspond to the respective *K*_M_ values (Michaelis constant) determined in *A. thaliana* leaf material with 200 μM uridine, 200 μM inosine, and 60 μM xanthosine. (b) Subcellular localization of URH1-GFP and URH2-GFP in the cytosol of transiently transformed *A. thaliana* protoplasts.

Ribonucleoside hydrolase activity was quantified in crude extracts of roots and leaves by determination of uracil, hypoxanthine or xanthine originating from added uridine, inosine or xanthosine, respectively. Further degradation of nucleobases was not observed, presumably because of missing cofactors in the enzyme assays. Evaluation of the *in vivo* kinetic properties of total soluble nucleosidase activities was performed with extracts of leaf tissue by applying increasing substrate concentrations until no further increase in reaction rate was observed. In this way, Michaelis constants (*K*_M_) of 200 μM for uridine and inosine and 60 μM for xanthosine were determined with a Lineweaver–Burk plot (Supporting Information Fig. S1). For all subsequent analyses, substrate concentrations at these *K*_M_ values were used. The highest total nucleosidase capacity was found in extracts of roots for all tested substrates. Less than 8 pkat mg^−1^ soluble proteins for each substrate was measurable in leaves (Fig. 1a).

The similar *in vivo K*_M_ values for the three tested substrates indicate comparable specificities of nucleoside hydrolases for their pyrimidine and purine substrates. In all cases, the determined *K*_M_ values were at least 40 times higher than the respective concentrations found in different plant tissues (Table 1). However, the observed *K*_M_ of 200 μM for uridine is still lower than that reported for mung bean (*Vigna radiata*), which is 1 mM (Achar & Vaidyanathan, 1967). Some legumes are suspected to have a higher nucleoside metabolism, as they produce secondary metabolites from pyrimidines (Brown & Turan, 1995). Nevertheless, the total capacity of uridine hydrolase in *A. thaliana* is comparable to that detected in these legume seedlings (53–82 pkat mg^−1^ protein).

**Table 1:** Metabolite content in roots and leaves of *Arabidopsis thaliana* wild-type and mutant plants

	Metabolite content (nmol g^−1^ fresh weight)						
	
	*URH*		*urh1*		*urh2*		*urh1urh2*
				
	Root	Leaf	Root	Leaf	Root	Leaf	Root
Uracil	1.0 ± 0.4	0.9 ± 0.5	1.5 ± 0.3	1.6 ± 0.3	1.1 ± 0.2	1.2 ± 0.1	3.8 ± 3.7
Uridine	4.6 ± 1.9	5.1 ± 2.5	223 ± 41*	16.8 ± 4.6*	4.3 ± 1.0	10.1 ± 4.9	191 ± 49*
UMP	10.3 ± 5.1	11.4 ± 2.3	14.4 ± 5.9	8.4 ± 1.9	10.5 ± 4.9	6.9 ± 4.0	21.1 ± 8.3
UDP	7.1 ± 3.1	50.7 ± 15.9	17.9 ± 3.7*	25.0 ± 2.7	6.9 ± 1.6	33.9 ± 4.6	13.5 ± 5.5
UTP	10.5 ± 1.6	13.5 ± 5.6	53.5 ± 2.2*	8.4 ± 1.1	11.15 ± 2.8	11.0 ± 1.6	38.8 ± 8.1*
UDPG	22.6 ± 23.1	45.8 ± 18.2	135 ± 37*	86.7 ± 4.5*	20.7 ± 11.3	79.6 ± 3.4	186 ± 7.7*
Hypoxanthine	–	–	–	–	–	–	–
Inosine	–	–	–	–	–	–	–
IMP	–	–	–	–	–	–	–
Xanthine	–	nd	–	nd	–	nd	–
Xanthosine	–	–	576 ± 194*	36.1 ± 7.9*	–	–	857 ± 232*
XMP	–	–	–	–	–	–	–

Metabolites were determined in extracts from 10-wk-old soil-grown *A. thaliana* plants. Data show mean ± SD (*n* = 3). Wild-type (*URH*) and mutant plants were grown in a randomized plot at the same time. Significant differences (*P* < 0.05), as determined using unpaired two-tailed *t*-tests, are marked with an asterisk. –, metabolites below the detection limit; nd, not determined because of insufficient separation from another unknown substance; *URH*, nucleoside hydrolase.

### Nucleoside hydrolase isoforms of *A. thaliana*

Using the protein sequences of the three ribonucleoside hydrolases RihA, RihB, and RihC of *E. coli* (Petersen & Møller, 2001) and the blastp algorithm, we identified no more than the same two isoforms in the nonredundant proteome of *A. thaliana* as Jung and co-workers found by performing a BLAST search with *Saccharomyces cerevisiae* URH1 (Kurtz *et al.*, 2002; Jung *et al.*, 2009). The genes are annotated as uridine ribohydrolases AtURH1 (At2g36310) and AtURH2 (At1g05620), and show only 49% sequence identity to each other. Both sequences contain typical features of the superfamily of nucleoside hydrolases (Versees & Steyaert, 2003; National Center for Biotechnology Information Conserved Domain Database (NCBI CDD) identifier: cd00455): the N-terminal cluster of Asp residues (DXDXXXDD motif), one additional conserved Asp residue and one conserved Thr residue for binding of cofactor Ca^2+^ (Degano *et al.*, 1996), and the His residue, which acts as a proton donor for the nucleobase product (Gopaul *et al.*, 1996) (Fig. S2). Standard prediction software classifies URH1 and URH2 as cytosolic proteins. This was confirmed by transformation of *A. thaliana* protoplasts with URH fusions to GFP, and subsequent detection of both proteins in the cytosol (Fig. 1b). Publicly available gene expression data from the AtGenExpress effort (Schmid *et al.*, 2005) show high abundances of both *URH* transcripts in root tissue (Fig. S3). This pattern closely reflects the distribution of uridine, inosine, and xanthosine nucleosidase activity measured in different organs of the *A. thaliana* plants (Fig. 1a).

One possible explanation for a relatively high abundance of nucleoside hydrolases in roots is that nucleosides are present in the soil and may represent a valuable nitrogen and carbon source for heterotrophic plant roots. Phillips *et al.* (1997) showed, for example, a high uridine content of *c.* 600 nmol g^−1^ in nonagricultural soil. This is 130 times higher than uridine contents in *A. thaliana* roots. With the help of equilibrative nucleoside transporters (Li *et al.*, 2003), the extracellular nucleosides may be taken up and used as substrates in salvage reactions in the cytosol of the root cells.

### Phenotypic analysis of all *URH* knock-out mutants

**Fig. 2:**
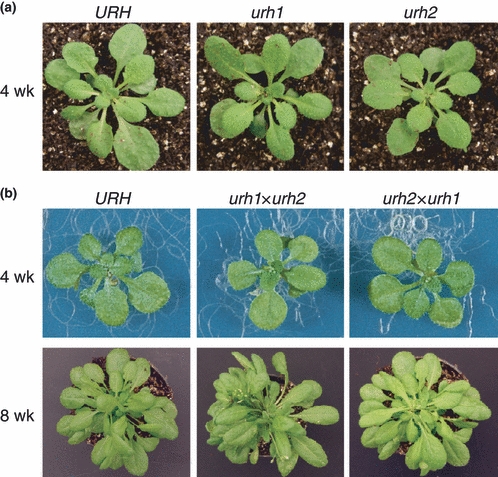
Phenotypical analysis of *Arabidopsis thaliana* wild-type and mutant plants. (a) Four-week-old wild-type (*URH*) and single mutant (*urh1* and *urh2*) plants grown on soil under an 8-h light regime. (b) Four-week-old wild-type (*URH*) and double mutant (*urh1*× *urh2* and *urh2*× *urh1*) plants grown on sterile nutrient agar under a 12-h light regime, and after transfer to soil for four more weeks under an 8-h light regime. *URH*, nucleoside hydrolase.

To assess the *in vivo* importance of uridine hydrolase, we used *A. thaliana* lines with T-DNA insertions in either the *URH1* or the *URH2* gene, which were selected for homozygosity. The T-DNA insertions were confirmed by sequencing flanking regions and absence of expression of the respective *URH* gene has been proved (Fig. S4). According to The Arabidopsis Information Resource (TAIR) Release 9, the T-DNA insertion in *URH1* is on chromosome 2, location 15226135, in the second intron of eight, several hundred bases downstream on the chromosome, as annotated. The T-DNA insertion in *URH2* is on chromosome 1, location 1680830, in the sixth intron of eight, as annotated. No visible phenotype was observed among homozygous single mutants when they were grown either aseptically on full nutrition medium or on soil (Fig. 2a). Also, homozygous *urh1* *urh2* and *urh2* *urh1* double mutants showed no visible differences in growth and development compared with wild-type *A. thaliana* (*URH*) (Fig. 2b).

In contrast to Jung *et al.* (2009), who reported delayed germination using an artificial microRNA (amiRNA) approach specific for *URH1*, we found no alterations in seedling germination of *urh1* and *urh2* single knock-out mutants, or in the *urh* double mutants. Furthermore, plant growth and development were unaffected in all knock-out lines compared with the wild type under standardized growth conditions (Fig. 2). As it was shown previously that *A. thaliana* miRNAs and also amiRNAs have a very narrow action spectrum and target only mRNAs with few mismatches (Schwag *et al.*, 2006), we can only speculate whether there are further effects in the 35S:amiAtURH1 transformants of Jung *et al*. Nevertheless, our careful screening of mutants with complete knock-out of both *URH* genes clearly demonstrates that neither URH1 nor URH2 represents a key regulator. Furthermore, the *urh* double mutants indicate that either nucleoside hydrolysis is metabolically unimportant or there are other enzymes present to take over the URH function.

Plant nucleosidases may also catalyze the conversion of cytokinin riboside to its active form, the cytokinin base (Chen & Kristopeit, 1981). However, studies in rice (*Oryza sativa*) indicate that gradients of active cytokinins are generated by phosphoribohydrolase activity (Kurakawa *et al.*, 2007). Furthermore, it has been shown that reduced active cytokinins cause enhanced root growth (Werner *et al.*, 2010). As plant growth and development were unaffected in all knock-out lines, a relationship between URH and cytokinin metabolism does not seem very likely.

### Nucleoside contents in *URH* knock-out mutants

To investigate the distribution of metabolites along the reaction chain from the nucleobases to nucleotide sugars, we quantified all intermediates in roots and leaves of wild-type and mutant plants using HPLC (Table 1). Nucleoside contents were low in wild-type tissues, with inosine and xanthosine concentrations below the detection limit. The uracil content in the wild type was 1 nmol g^−1^ fresh weight, while hypoxanthine and xanthine were below the detection limit. The predominant nucleotides were UDP glucose (UDPG) and the multiple phosphorylated nucleosides UDP and UTP, while IMP and XMP were below the detection limit. All other adenine and guanosine intermediates (nucleotides, nucleosides and nucleobases) were unchanged and are not shown. Together with uracil, uridine contributed to < 10% of the total soluble pyrimidine pool in the organs examined. One possible reason for the low contents of nucleosides and nucleobases is active salvage to channel them directly to pools of phosphorylated pathway intermediates; another is active degradation. In addition, many nucleotides arising from nucleic acid turnover (UMP) and carbohydrate synthesis (UDP) may not attain the unphosphorylated state, but are readily rephosphorylated by several kinases (Zhou *et al.*, 1998; Lange *et al.*, 2008).

When compared with the wild-type, there was a highly significant enlargement of the uridine pool in the roots of *urh1* mutants (Table 1). Also, pool sizes of other pyrimidine metabolites increased significantly. The second predicted substrate of this class of nucleoside hydrolase, inosine, did not accumulate. However, the content of another purine nucleoside, xanthosine, increased substantially to pool sizes of 576 nmol g^−1^ fresh weight in the roots of *urh1*. None of the measured intermediates accumulated in *urh2* mutants (Table 1), while roots of double mutants showed the same accumulation pattern as *urh1* mutants (Table 1). As metabolism of purines and that of pyrimidines follow distinct pathways, the xanthosine accumulation was probably not a consequence of pyrimidine accumulation but of impaired xanthosine hydrolysis. These findings clearly demonstrate that xanthosine also has to be considered as a substrate of plant enzymes annotated as uridine hydrolases.

### Nucleoside hydrolytic activities in *URH* knock-out mutants

**Fig. 3:**
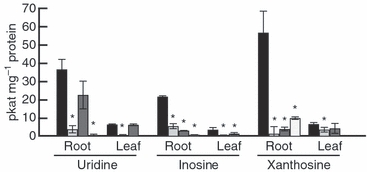
Nucleoside hydrolytic activities in *Arabidopsis thaliana* wild-type and mutant plants. Nucleoside hydrolysis was determined in soluble fractions of plant extracts. Data show mean ± SD (*n*= 3). Significant differences (*P* < 0.05) between mutant (*urh1*, pale gray bars; *urh2*, dark gray bars; *urh1 × urh2*, white bars) and wild-type (*URH*, black bars), as determined using unpaired two-tailed *t*-tests, are marked with an asterisk. *URH*, nucleoside hydrolase.

Nucleoside hydrolytic activities were measured with uridine, inosine and also xanthosine as substrates. Uridine hydrolase capacity was significantly reduced in soluble extracts of roots and leaves of *urh1,* thus confirming its role as the major uridine nucleosidase in *A. thaliana*. Loss of URH2 did not significantly affect the uridine hydrolytic capacity of roots or leaves of *A. thaliana* plants, while in double mutants almost no uridine hydrolytic activity could be found in roots (Fig. 3).

When the purine nucleosides inosine and xanthosine were used as substrates, both hydrolase activities were considerably decreased in soluble extracts of each of the mutants (Fig. 3). This shows, for the first time, that URH1 is not only needed for uridine degradation, but may also be directly involved in purine hydrolysis. This also indicates that URH2 has to be considered as a purine hydrolase rather than a uridine hydrolase. In addition, the remaining nucleoside hydrolytic capacity in roots of the double mutants (Fig. 3) suggests the existence of another as yet unidentified hydrolase. As already discussed (Jung *et al.*, 2009), there is one other gene coding for a putative functional nucleoside hydrolase present in the *A. thaliana* genome (*At5g18860*). This gene probably codes for a dual domain protein whose translation product was found in the cell wall proteome. Thus it is more likely to be a candidate for the cell wall-bound adenosine nucleosidase (Riewe *et al.*, 2008), that would be found in the residue of our enzyme extract.

### Nucleoside uptake and metabolism in *URH* knock-out mutants

**Fig. 4:**
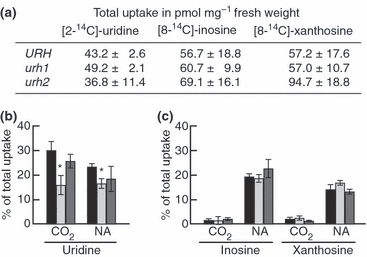
Nucleoside uptake and metabolism in wild-type and mutant plants. (a) Total uptake after feeding ^14^C-nucleosides to roots of *Arabidopsis thaliana* plants. Data show mean ± SD (*n* = 2–4). (b) ^14^CO_2_ formation and ^14^C incorporation into nucleic acids (NA) after feeding [2-^14^C]-uridine to roots of *A. thaliana* plants. Data show mean ± SD (*n*= 4). Significant differences (*P* < 0.05) between mutants (*urh1*, pale gray bars*; urh2*, dark gray bars) and wild-type (*URH*, black bars), as determined using unpaired two-tailed *t*-tests, are marked with an asterisk. (c) ^14^CO_2_ formation and ^14^C incorporation into nucleic acids (NA) after feeding [8-^14^C]-inosine and [8-^14^C]-xanthosine to roots of *A. thaliana* plants. Data show mean ± SD (*n* = 2–4). No significant differences (*P* < 0.05) between mutants (*urh1*, pale gray bars; *urh2*, dark gray bars) and wild-type (*URH*, black bars) were found. *URH*, nucleoside hydrolase.

To further assess the *in vivo* importance of soluble nucleoside hydrolytic activities, feeding studies with [2-^14^C]-uridine, [8-^14^C]-inosine and [8-^14^C]-xanthosine were performed in the wild type and mutants. The plants were grown aseptically on solid medium, and nucleosides were administered to the roots to closely resemble natural situations. Radioactivity of all used substrates was readily incorporated at comparable rates (Fig. 4a). This was probably attributable to the uptake system itself, as nucleoside transporters are present and accept purine and pyrimidine substrates (Li *et al.*, 2003; Wormit *et al.*, 2004). Although uridine and xanthosine were increased in all organs of *urh1* mutant plants (Table 1), there was no significant difference in uptake. This shows that augmented nucleoside contents do not restrict further uptake. It may also hint at sequestration of nucleosides in different cellular compartments; for example, the vacuole. Analyses of subcellular metabolite accumulation would clarify this, but this question is not addressed in the present analysis. Additionally, in the studies carried out, we cannot exclude the possibility of extracellular nucleoside hydrolysis, and the possibility that unlabeled ribose and the respective nucleobase were taken up and further metabolized.

When uridine was fed to *A. thaliana* plant roots, *c.* 30% of the uptake was degraded and released as CO_2_, while *c.* 20% was salvaged and integrated into the nucleic acid fraction (Fig. 4b). Comparison of the wild type with mutant plants revealed a significant reduction of CO_2_ production from fed uridine in *urh1* mutants. While uridine catabolism to CO_2_ in *urh1* was reduced by half, no changes were observed in *urh2* single mutants, and also no further significant reduction of uridine catabolism below the level of *urh1* was found in the double mutants (46% of the wild-type level). Thus, the remaining capacity for uridine hydrolysis in the double mutants was sufficient to accomplish 46% of uridine degradation under *in vivo* conditions. This clearly demonstrates that neither URH is a key regulator of uridine degradation.

The increased pool sizes of uridylates and UDP glucose in roots of *urh1* mutants (Table 1) indicate enhanced uridine salvage, starting with uridine kinase and followed by further phosphorylations. These increased pools might be the reason for the presumably reduced incorporation into nucleic acids in the *urh1* mutant (Fig. 4b), but it is more likely a consequence of diluting effects and reduced specific activity in the respective pools.

When inosine and xanthosine were fed to *A. thaliana* plant roots, *c.* 19% of the inosine uptake and an astonishing 14% of the xanthosine uptake were integrated into the insoluble fraction (Fig. 4c). As they were supplied as [8-^14^C]-inosine and [8-^14^C]-xanthosine, the label in the insoluble fraction can be regarded as salvaged nucleotides that are integrated into nucleic acids. To date, incorporation of xanthosine into nucleic acids has not been found in potato (*Solanum tuberosum*) tuber slices (Katahira & Ashihara, 2006), leaves of cacao (*Theobroma cacao*) (Koyama *et al.*, 2003), or tea seedlings (*Camellia sinensis*) (Deng & Ashihara, 2010), but it should be kept in mind that previous feeding studies always used excised tissue, whereas our set-up resembled the natural situation. Further separation of the insolubles revealed main labeling in the KOH hydrolysable fraction, which is RNA. This shows for the first time that *A. thaliana* plants are able to salvage xanthosine, at least to a certain extent.

In contrast with [2-^14^C]-uridine, [8-^14^C]-inosine and [8-^14^C]-xanthosine degradation does not entail immediate ^14^CO_2_ release. In fact, radioactivity will remain in soluble degradation intermediates such as allantoate, ureidoglycine and ureidoglycolate, and only from a very small proportion of these is ^14^CO_2_ produced (Fig. 4c). These differences in nucleoside metabolism between purines and pyrimidines may be a result of their partially different functions in the plant. While both are building blocks of nucleic acids, an active and efficient recycling of the purine adenosine is very important for primary metabolism in transmethylation reactions.

Inosine and xanthosine metabolism was unchanged in both *urh1* and *urh2*, while the levels of degradation to CO_2_ were fairly low (2% of uptake). It is known from a few plant studies that purine degradation may occur via the formation of xanthosine, either by deamination of guanosine or by dephosphorylation of XMP, and subsequent hydrolysis to xanthine. Xanthine may also be built from hypoxanthine or guanine (Fig. S5). The relative contributions of these reactions to salvage and degradation depend on the analyzed material (Ashihara *et al.*, 1997; Katahira & Ashihara, 2006). As inosine and xanthosine hydrolytic capacity is greatly reduced in soluble protein extracts of *urh1* and *urh2* mutants and only xanthosine accumulates, these plants presumably perform active inosine salvage and purine degradation by guanine deaminase. As *urh* mutants are able to metabolize inosine and xanthosine and do not show phenotypic differences, no substantial reduction of purine degradation is assumed, as this would cause visible changes in growth and development (Nakagawa *et al.*, 2007).

These results strongly suggest the presence of other enzymes to take over URH function, such as the above-mentioned cell wall-bound adenosine nucleosidase (Riewe *et al.*, 2008). Therefore, analysis of nucleoside hydrolytic activities in the residue of the enzyme extracts of roots was performed. As evident from Table 2, there was some reduction of uridine and xanthosine nucleosidase activities in the residues of both *urh* mutant plants. In addition to a very high inosine nucleosidase capacity, xanthosine and uridine could also still be hydrolyzed from residual enzyme activities present in the pellet fraction of enzyme extracts. Whether these activities were attributable to the existence of the above-mentioned candidate will be the subject of further studies using a combination of different double and triple mutants.

**Table 2:** Nucleoside hydrolase activity in root extract pellets of *Arabidopsis thaliana* wild-type and mutant plants

	Nucleoside hydrolase activity (pkat g^−1^ fresh weight)				
	
	Uridine	Inosine	Xanthosine	Adenosine	Guanosine
*URH*	2.35 ± 0.56	344.79 ± 43.19	23.22 ± 0.60	47.78 ± 15.67	214.96 ± 15.60
*urh1*	0.45 ± 0.44*	519.49 ± 150.89	13.51 ± 6.59*	154.42 ± 57.55*	236.17 ± 22.68
*urh2*	2.85	525.68 ± 157.47	13.97 ± 9.13	100.94 ± 36.47	200.78 ± 16.72

Activities were determined in pellets of extracts from 10-wk-old soil-grown *A. thaliana* plants. Data show mean ± SD (*n* = 3). Wild-type (*URH*) and mutant plants were grown in a randomized plot at the same time. Significant differences (*P* < 0.05), as determined using unpaired two-tailed *t*-tests, are marked with an asterisk. *URH*, nucleoside hydrolase.

### Both URH1 and URH2 are required for efficient inosine and xanthosine hydrolytic activity

**Fig. 5:**
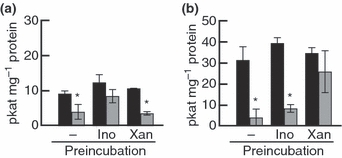
Recovered inosine hydrolase (a) and xanthosine hydrolase (b) activity of *Arabidopsis thaliana* nucleoside hydrolase (*urh*) mutant extracts. *URH*, black bars; *urh1 + urh2*, gray bars. Soluble root extracts from *urh1* and *urh2* mutants were mixed and preincubated for 1 h with substrate (−, no substrate; Ino, inosine; Xan, xanthosine) at the respective *K*_M_ (Michaelis constant) concentration. Substrates were removed by desalting on spin columns using Sephadex G-25 fine before activity measurement. Data show mean ± SD (*n* = 2–3). Significant differences (*P* < 0.05) between mutant mix (*urh1 + urh2*) and wild-type (*URH*), as determined using unpaired two-tailed *t-*tests, are marked with an asterisk.

As shown previously, inosine and xanthosine hydrolysis is strongly reduced in soluble extracts of *urh1* and *urh2* mutants. Because the remaining activities in the roots of the *urh* mutants do not sum to the activity in the wild type, neither for the substrate inosine nor for xanthosine, it was postulated that both URH1 and URH2 proteins are necessary for inosine and xanthosine hydrolysis. To test this hypothesis, mutant extracts were mixed and incubated with or without substrate before enzyme activity measurements. Combining both mutant extracts with the respective substrate restored inosine or xanthosine hydrolytic activity (Fig. 5), thus revealing the necessity of at least these two URH isoforms for active inosine and xanthosine hydrolysis in soluble protein extracts of plants. This conclusion is further supported by *in vitro* activity reconstitution using a mixture of recombinant URH1 and URH2 proteins, expressed in *E. coli* after Ni-NTA purification (Fig. S6). However, persistent formation of inclusion bodies and hence a low yield of recombinant soluble URH2 prevented us from carrying out a more rigorous biochemical characterization of the recombinant protein complex. It also has to be considered that the exact stoichiometry of the postulated URH1–URH2 heteromers is not known, and the potential activity of the heteromers in inosine and xanthosine hydrolysis may have been underestimated in our *in vitro* data. Furthermore, our data also indicate the involvement of additional proteins or regulatory processes for modulation of catalytic activity *in vivo*, because *urh2* mutants were strongly compromised in xanthosine hydrolytic activity (Fig. 3), although the recombinant URH1 protein alone exhibited xanthosine hydrolysis (Fig. S6).

To date, the occurrence as multimers of identical proteins has been demonstrated for several nucleoside hydrolases of protists (Parkin, 1996), but the existence of heteromeric complexes has not been shown in plants (Guranowski, 1982; Szuwart *et al.*, 2006). However, the coexistence of homomeric and heteromeric complexes of different isoforms with distinct functions has been shown for other enzymes; for example, aldehyde oxidases in *A. thaliana* (Akaba *et al.*, 1999). In addition, it is assumed that substrate specificity of nucleosidases is not absolutely strict, and substrate recognition seems to be mainly based on the ribose moiety (Versees & Steyaert, 2003). On the basis of our metabolite and activity data, we propose that there are nucleosidase enzyme complexes in plants with different subunit compositions, which determine their substrate specificities. In the case of *A. thaliana*, an URH1 homomer may be responsible for uridine hydrolysis, while an URH1–URH2 heteromer is responsible for xanthosine and inosine hydrolysis.

This theory is further supported by the fact that at least one homolog of each of the two *URH* genes can be found in the completely sequenced genomes of moss (*Physcomitrella patens*), monocotyledons (*Zea mays*, *Sorghum bicolor* and *Oryza sativa*), and dicotyledonous species (*Populus trichocarpa*, *Vitis vinifera* and *A. thaliana*). The occurrence of both isoforms in the Embryophyta genomes suggests a preservation of the URH1–URH2 functional relationship during evolution.

### Conclusion

This investigation of plant mutants identified the hitherto superficially investigated xanthosine as a central substrate for cytosolic nucleoside hydrolases. Our findings revealed that nucleoside hydrolase activity and substrate specificity also depend on the presence and interaction of different subunits. To date, only heterologously expressed single proteins have been used for such analyses, including crystallization studies (Petersen & Møller, 2001; Versees & Steyaert, 2003; Kim *et al.*, 2006; Iovane *et al.*, 2008). Therefore, standard *in vitro* analysis was unsuitable for the detection of this phenomenon, concomitantly demonstrating the utility of cautious *in planta* biochemical analysis. These results are also of great significance for investigations of purine alkaloid biosynthesis in caffeine- and theobromine-producing plants, especially as xanthosine is the starting metabolite for this class of secondary plant products, and we present a way of achieving very substantial accumulation of this precursor.

In addition, double mutants of the hitherto identified nucleoside hydrolases suggest the involvement of additional as yet unidentified genes in plant nucleoside hydrolysis. In addition to the detection of a potential candidate gene by sequence comparison, our activity measurements clearly show another nucleoside hydrolase activity with a different substrate profile, spatially separated from the two herein described. In-depth analysis of this promising candidate will be the subject of further studies using double and triple mutants.

## Supporting Information

**Fig. S1** *In vivo* kinetic properties of nucleoside hydrolase activity in *Arabidopsis thaliana* leaf material.

**Fig. S2** Multiple sequence alignment of three ribonucleoside hydrolases, RihA, RihB, and RihC, of *Escherichia coli* and the two nucleoside hydrolase (URH) isoforms of the nonredundant proteome of *Arabidopsis thaliana* using Clustal 2.0.12.

**Fig. S3** RNA expression of nucleoside hydrolase 1 (*URH1; At2g36310*) and *URH2 (At1g5620)* according to Genevestigator.

**Fig. S4** Expression of nucleoside hydrolase (*URH*) genes in *urh* mutants.

**Fig. S5** Nucleoside and nucleobase salvage pathways for purines and pyrimidines.

**Fig. S6** Nucleoside hydrolase activity of recombinant nucleoside hydrolase (URH) proteins.
