# Supplementary material for: Arabidopsis thaliana nucleosidase mutants provide new insights into nucleoside degradation
Source: New Phytol. 2011 Jul;191(2):349–59. doi: 10.1111/j.1469-8137.2011.03711.x (PMC3147060; doi:10.1111/j.1469-8137.2011.03711.x)
Supplement: Supplementary file 1 [file nph0191-0349-SD1.pdf]

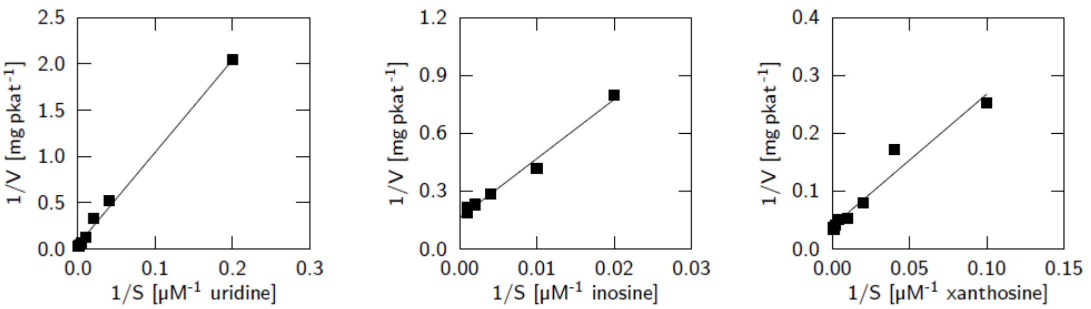

Figure S1. In vivo kinetic properties of nucleoside hydrolase activity in Arabidopsis leaf material. Data represent means of two biological replicates. Kinetic constants were determined from Lineweaver-Burk plots as follows: uridine ribohydrolase K_M = 202,5 µM, inosine ribohydrolase K_M = 206,8 µM, xanthosine ribohydrolase K_M = 57,9 µM.

```
                            #   -#                        -
RihA            ------------------MALP-ILLDCDPGHDDAIAIVLALASPELDVKAITSSAGNQ 40
RihB            ------------------MEKRKIILDCDPGHDDAIAIMMAAKHPAIDLLGITIVAGNQ 41
RihC            ------------------MRLP-IFLDTDPGIDDAVAIAAAIFAPELDLQLMTTVAGNV 40
URH1            MDCGMENCNGGISNGDVLGKHEKLIIDTDPGIDDSMAILMAFQTPELEILGLTTVFGNV 59
URH2            ---------------MAIGDRKKIIIDTDPGIDDAMAIFVALNSPEVDVIGLTTIFGNV 44
                                       :::* *** **::**  *   * :::  :*   **

                                                          ++
RihA            TPEKTLRNVLRMLTLLNRTDIPVAGGAVKP--LMRELIIADNVHGESGLDGPALPEPTF 97
RihB            TLDKTLINGLNVCQKL-EINVPVYAGMPQP--IMRQQIVADNIHGETGLDGPVFEPLTR 97
RihC            SVEKTTRNALQLLHFW-NAEIPLAQGAAVP--LVRAPRDAASVHGESGMAGYDFVEHNR 96
URH1            STQDATRNALLLCEIAGFPDVPVAEGSSEP-LKGGIPRVADFVHGKNGLGDVSLPPPSR 117
URH2            YTTLATRNALHLLEVAGRTDIPVAEGTHKTFLNDTKLRIADFVHGKDGLGNQNFPPPKG 103
                    :  * * :       ::*:  *   .         *  :**: *: .  :   .

                                      #
RihA            APQNCTAVELMAKTLRESAEPVTIVSTGPQTNVALLLNSHPELHSKIARIVIMGGAMGL 155
RihB            QAESTHAVKYIIDTLMASDGDITLVPVGPLSNIAVAMRMQPAILPKIREIVLMGGAYGT 155
RihC            KPLGIPAFLAIRDALMRAPEPVTLVAIGPLTNIALLLSQCPECKPYIRRLVIMGGSAGR 154
URH1            KKSEKSAAEFLDEKVEEYPGEVTILALGPLTNLALAIKRDSSFASKVKKIVILGGAFFS 176
URH2            KPIEKSGPEFLVEQAKLCPGEITVVALGPLTNLALAVQLDPEFSKNVGQIVLLGGAFAV 162
                      .   : .        :*::. ** :*:*: :   .     : .:*::**:

                  -     -+-
RihA            -GNWTPAAEFNIYVDPEAAEIVFQSGIPVVMAGLDVTHKAQIHVEDTERFRAIGNPVST 214
RihB            -GNFTPSAEFNIFADPEAARVVFTSGVPLVMMGLDLTNQTVCTPDVIARMERAGGPAGE 214
RihC            -GNCTPNAEFNIAADPEAAACVFRSGIEIVMCGLDVTNQAILTPDYLSTLPQLN-RTGK 212
URH1            LGNVNPAAEANIYGDPEAADVVFTSGADITVVGINITTQLKLSDDDLLELGNCKGKHSK 235
URH2            NGNVNPASEANIFGDPEAADIVFTCGADIIAVGINVTHQVIMTADDKDKLASSKGKLAQ 221
                 ** .* :* ** *****  ** .*  :   *:::* :     :    :       .

                        +   +            +#
RihA            IVAELLDFFLEYHKDEKWGFVGAPLHDPCTIAWLLKPELFTSVERWVGVETQGKYTQGM 273
RihB            LFSDIMNFTLKTQFENY-GLAGGPVHDATCIGYLINPDGIKTQEMYVEVDVNSGPCYGR 272
RihC            MLHALFSHYRSGSMQS-----GLRMHDLCAIAWLVRPDLFTLKPCFVAVETQGEFTSGT 266
URH1            LISDMCKFYRDWHVKSD-GVYGVYLHDPVSFVAVVRPDLFTYKKGVVRVETQG-ICVGH 292
URH2            YLCKILDVYYDYHLTAY-EIKGVYLHDPATILAAFLPSLFTYTEGVARVQTSG-ITRGL 278
                 .  : .   .          *  :**   :   . *. :.     . *:...    *

RihA            TVVDYYYLTGNKPN-------ATVMVDVDRQGFVDLLADRLKFYA--- 311
RihB            TVCDELGVLGKPAN-------TKVGITIDTDWFWGLVEECVRGYIKTH 313
RihC            TVVDIDGCLGKPAN-------VQVALDLDVKGFQQWVAEVLALAS--- 304
URH1            TLMDQGLKRWNGSNPWVGYSPISVAWTVDVEGVLEYVKAKLMKP---- 336
URH2            TLLYNNLKRFEEANEWSDKPTVKVAVTVDAPAVVKLIMDRLMES---- 322
                *:        : .*         *   :*   .   :   :
```

Figure S2. Multiple sequence alignment of three ribonucleoside hydrolases RihA, RihB, and RihC of *Escherichia coli* and the two URH isoforms of the nonredundant proteome of Arabidopsis using Clustal 2.0.12. Symbols below the alignment are as follows: *, identical residue; :, conserved substitution; ., semiconserved substitution. Symbols above the alignment indicate conserved residues involved in binding: #, Ca^{2+} binding; -, ribose binding; +, base binding.

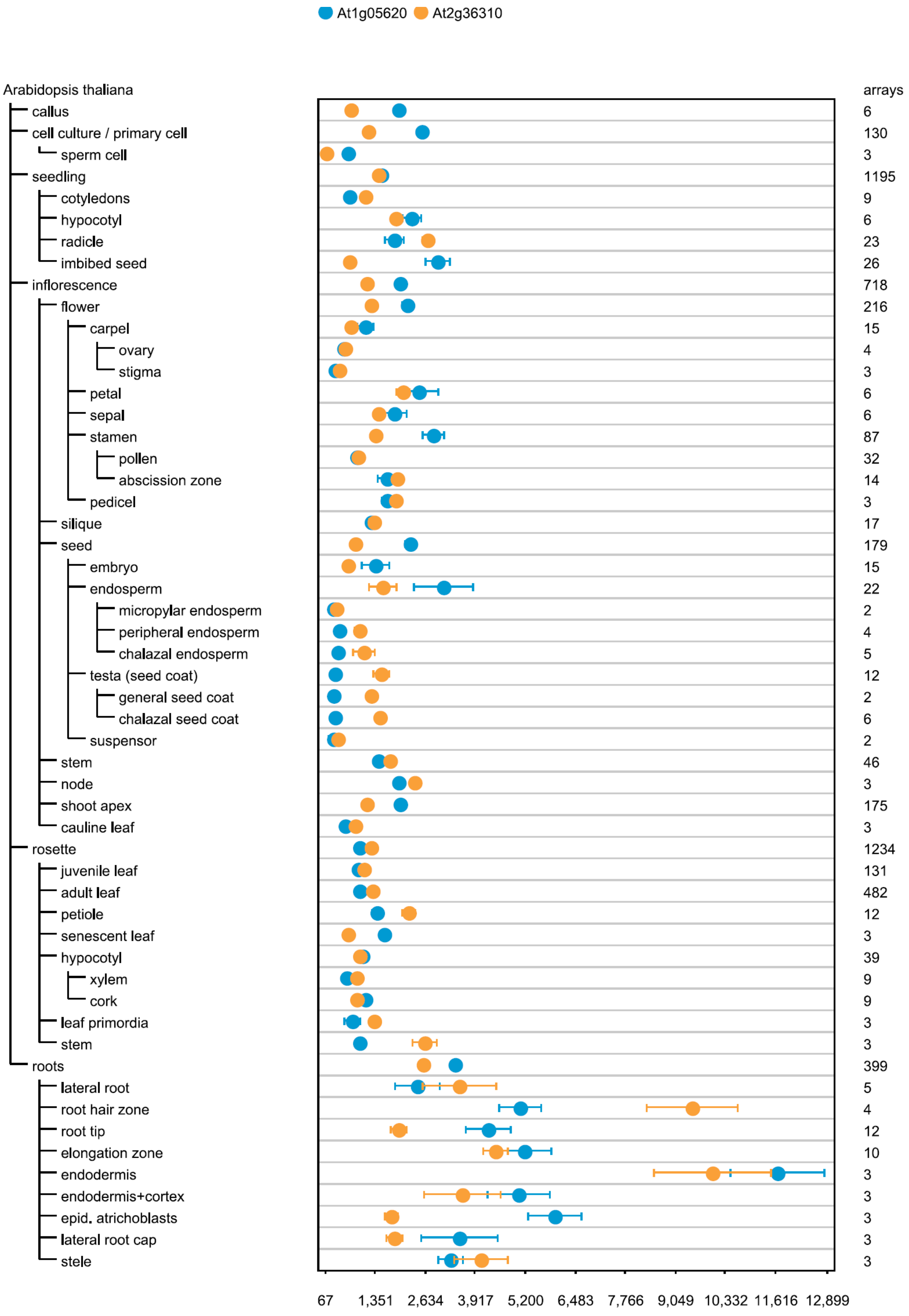

Figure S3. RNA expression of *URH1* (At2g36310) and *URH2* (At1g5620) according to Genevestigator (**Zimmermann P, Hirsch-Hoffmann M, Hennig L, Gruissem W.** (2004) GENVESTIGATOR: Arabidopsis microarray database and analysis toolbox. Plant Physiol **136**: 2621-2632).

A

	5' region	3' region
URH	0.011596	0.029743
urh1	0.000006	0.000601

	oligonucleotide sequence
URH1 5' F	5'-AGACCCAGGAATTGATGATAGC-3'
URH1 5' R	5'-CGTTGCGAGTAGCATCTTGT-3'
URH1 3' F	5'-ACTATCAGATGATGACCTCTTGGAG-3'
URH1 3' R	5'-TTTGACGTGCCAATCTCTATAAAA-3'
EF 1α 5' F	5'-TGGTGACGCTGGTATGGTTA-3'
EF 1α 5' R	5'-TCCTTCTTGTCCACGCTCTT-3'

B

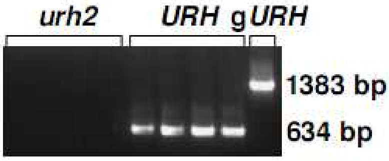

	oligonucleotide sequence
URH2 F	5'-TTCACCTGAAGTTGATGTCATTG-3'
URH2 R	5'-TTTCGTAAGCCGTGAGATGA-3'

Figure S4. Expression of *URH* genes in *urh* mutants. A, Relative Expression of *URH1* in *urh1* mutant and wildtype Arabidopsis. The transcript levels were determined by quantitative realtime RT-PCR using two gene specific primer pairs in the 3' or 5' part of the transcript. The transcript levels were calculated relative to elongation factor 1α. Data show means of four biological replicates. B, RT-PCR expression analysis of *URH2* transcript (634bp fragment) in *urh2* mutant and wildtype Arabidopsis (*URH*). RNA was extracted from 4 biological replicates of *urh2* mutant and wildtype Arabidopsis and processed as described in the Methods section. Genomic DNA of wildtype Arabidopsis (*gURH*) was used as control. All plants used in this analysis were six weeks old.

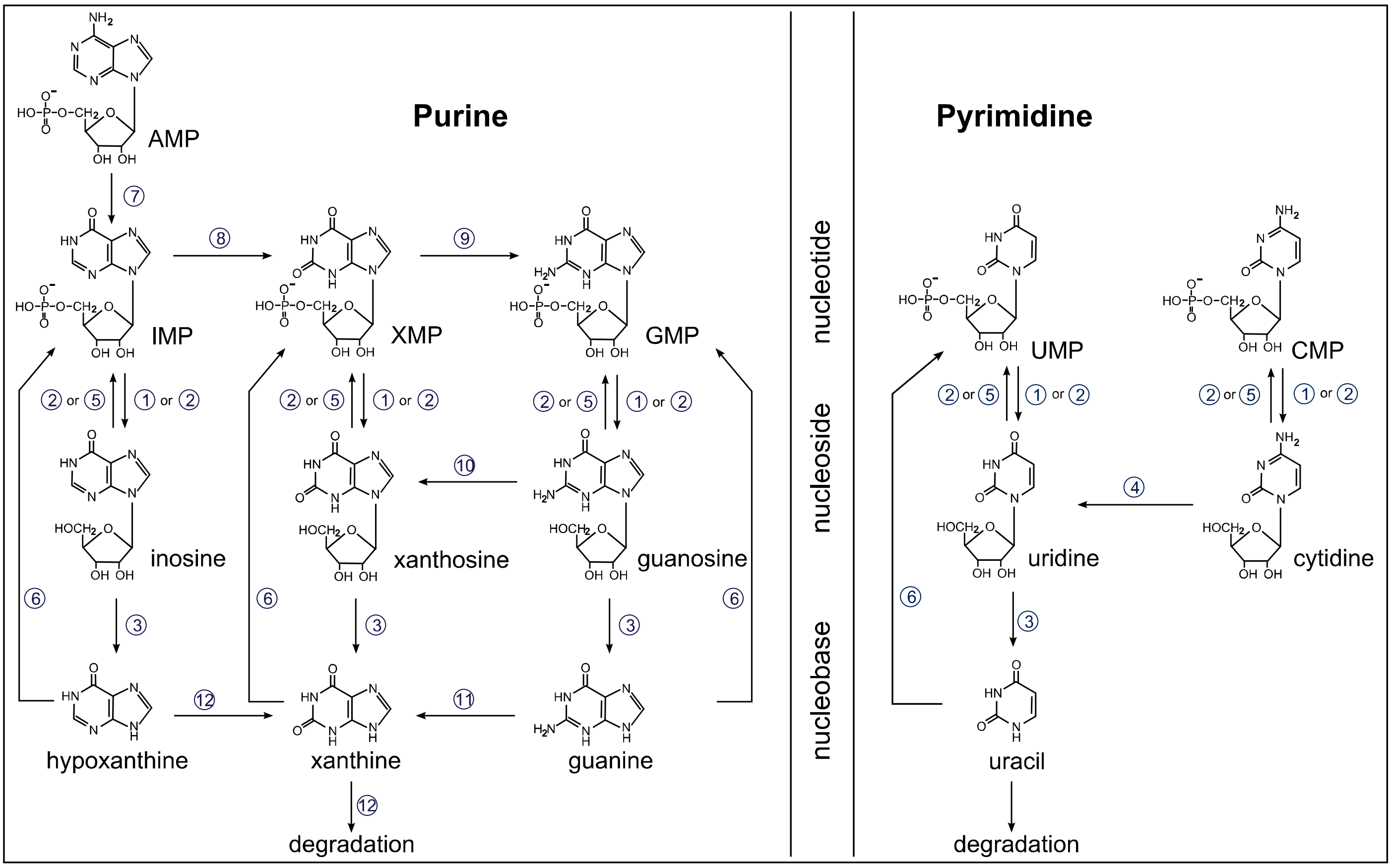

Figure S5. Nucleoside and nucleobase salvage pathways for purines and pyrimidines. Enzymes are numbered and general enzyme nomenclature is as follows: 1) NMP specific 5' nucleotidase; 2) nucleoside phosphotransferase; **3) nucleoside hydrolase**; 4) cytidine deaminase; 5)nucleoside kinase; 6) nucleobase phosphoribosyl transferase; 7) AMP deaminase; 8) IMP dehydrogenase; 9) GMP synthase; 10) guanosine deaminase; 11) guanine deaminase; 12) xanthine dehydrogenase

A

	enzyme activity in pkat mg^{-1} protein		
	uridine	inosine	xanthosine
URH1	7279.2 ± 59.3	982.2 ± 171.6	2134.6 ± 21.6
URH2	40.9 ± 0.5	45.2 ± 8.7	32.8 ± 2.2

B

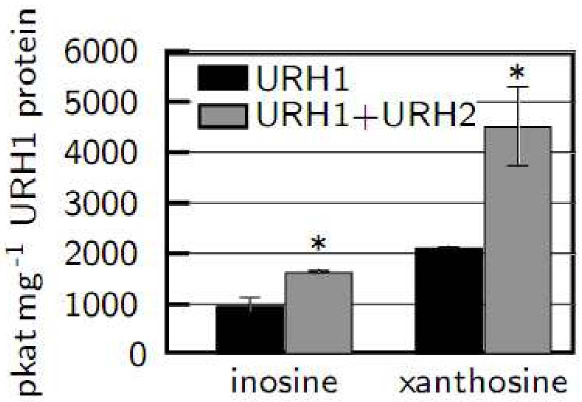

Figure S6. Nucleoside hydrolase activity of recombinant URH proteins. A, Total activity of nucleoside to nucleobase conversion after over-expression in *E. coli* and non-denaturing purification of recombinant URH1 and URH2 proteins from the soluble extracts. Activity measurements were performed from desalted eluate fractions with the respective nucleoside at 200 µM. Data are expressed in pkat per mg protein after Ni-NTA agarose purification and show means +/- SD (n = 3). B, Total activity of purified recombinant URH1 and a mixture of equal amounts of recombinant URH1 and URH2 from desalted eluate fractions. Data show means +/- SD (n = 3). Significant differences ($P < 0.05$) between URH1 and URH1 + URH2 using unpaired two-tailed *t*-tests are marked with *.
